# Impact of Consumption of Bananas on Attraction of *Anopheles stephensi* to Humans

**DOI:** 10.3390/insects9040129

**Published:** 2018-09-28

**Authors:** Susan Paskewitz, Patrick Irwin, Nic Konwinski, Scott Larson

**Affiliations:** Department of Entomology, University of Wisconsin, Madison, WI 53706, USA; pirwin@nwmadil.com (P.I.); nkonwinski12@gmail.com (N.K.); slarson@mmcd.org (S.L.)

**Keywords:** diet, host attraction, *Anopheles gambiae*, mosquito

## Abstract

Humans vary in attractiveness to mosquitoes, a phenomenon that is largely attributed to differences in physical cues such as heat and volatile odors emanating from breath and skin. Diet can change human odors but whether specific dietary components alter host attractiveness is largely unexplored. We identified bananas as a target for study following a survey of the internet for advice on avoiding mosquito bites. Human attractiveness to *Anopheles stephensi* Liston was measured using a glass vial bioassay where mosquito contacts were measured before and 1–3 h after ingestion of bananas or grapes. Consumption of grapes had no effect on the number of contacts but banana ingestion resulted in a significant increase in the overall number of contacts in spite of individual variation that included some subjects who showed no effect or decreases in contacts. Further tests with a single volunteer showed that the effect was repeatable and consistent across 15 trials. The magnitude of the increase was not affected by the number of bananas eaten. Increased contact counts after banana ingestion were also observed when *A. gambiae* Giles was tested. These results support the hypothesis that diet plays an important role in mediating host attractiveness to anopheline mosquitoes.

## 1. Introduction

In recent years, the mechanisms used by adult female mosquitoes to find and select hosts have come under intense study. Mosquitoes respond to host odors and our knowledge of the sensory apparatus for detecting and integrating these cues has increased dramatically [1,2,3,4,5]. Characterization of human odor profiles has provided an explanation for the observation that some people are more attractive to mosquitoes than others [6]. Some individuals emit chemical signals that elicit activation and arrestment responses in mosquitoes while others either emit less of these cues or emit chemicals that actually interfere with such responses [7,8]. Identifying both types of chemical cues as well as the sources of variation in these cues holds great promise for the development of novel methods for reducing the risk of vector-borne disease.

Many things can cause variations in human odor that alter mosquito attraction. These include genetics [9], the microbiota associated with skin [10], exercise [11,12], pathological conditions [13], age and sex [12], reproductive status [14,15,16], and externally applied products [17]. Diet also can cause variation in human odors [18,19]. However, only one dietary component has been clearly linked to mosquito attraction: the consumption of beer increases the attractiveness of body odors of subjects to both *Aedes albopictus* (Skuse) [20] and *Anopheles gambiae* Giles [21]. Other connections between diet and host attraction have not been assessed, but these interactions could affect disease transmission by altering patterns of mosquito biting among available hosts.

Interestingly, dietary components are frequently noted by the general public and non-specialists as affecting mosquito biting rates. Electronic searches for information utilizing web-based search engines and the keywords “diet and mosquito” identified several recurrent messages. Consumers are often advised that ingestion of garlic or vitamin B provides protection against mosquitoes. However, controlled tests of garlic pills and vitamin B12 showed no impacts on mosquito biting or landing rates [22,23]. Conversely, there are many recommendations to avoid eating certain foods as a way to reduce mosquito bites. These recommendations suggest that eating potassium-rich foods, salty snacks, spicy foods, and sweets will enhance attractiveness. Some foods, especially bananas, are variously reported to enhance and to decrease attraction, reflecting the confusion around this topic. To the best of our knowledge, none of these foods have been tested in controlled settings, so there is no evidence on which to base expert advice.

Here we report the results of a study to test whether banana ingestion affects the attraction of *Anopheles stephensi* Liston and *A. gambiae* to volatile odor cues transferred from human skin to a glass substrate. The two anopheline species were chosen because they are important malaria vectors and because anopheline mosquitoes were used in studies of the impact of alcohol [21] and vitamin B12 supplementation [22].

## 2. Materials and Methods

Mosquito rearing. *Anopheles stephensi* and *A. gambiae* adult females were held in 3.79 L (1 gallon) paper or plastic containers in incubators maintained at 26 ± 2 °C and 70–80% relative humidity, with constant access to 10% sucrose. Larval and pupal mosquitoes were reared in the insectary under the same temperature conditions. Larvae were fed a 2:1 mixture of ground fish food (Vita-pro plus) and brewer’s yeast. Adults were fed on purchased blood (defibrinated rabbit blood, Hemostat Laboratories, Dixon, CA, USA) using an artificial membrane feeder for colony maintenance.

Experimental design. Experiments were performed with bananas (Cavendish; *Musa acuminate* (L.)) and green grapes (Thompson; *Vitis vinifera* L.) purchased from local grocery stores. Bananas varied in degree of ripeness at the time of consumption from green-yellow to yellow with few black spots.

To test whether attractiveness might change following ingestion of a banana, we used a bioassay in which human skin compounds are transferred to a glass vial when rubbed by the subjects. This procedure eliminates the need to expose subjects to mosquito bites [7,22,24,25]. Subjects rolled a capped, clean glass vial (6.4 × 1.7 cm) between their hands for 2 min. Vials were prefilled with distilled water at 37 °C to simulate human body temperature and to increase volatilization of transferred compounds [22]. The vial was rapidly transferred to a 1 gal plastic carton (17.4 cm tall by 17.2 cm diameter) containing 20 naïve, colony-raised female mosquitoes. The warmed vial was placed on the floor of the carton and positioned vertically.

Mosquitoes were at rest on the sides of the carton or in flight as a result of the introduction of the vial at the start of the trial. The number of mosquitoes contacting the glass vial during a 2 min period was counted by the test subject. A “contact” was defined as touching the tube rather than landing on the tube with the proboscis or legs to simplify counting. Individual mosquitoes could not be discriminated so that a single mosquito might “contact” multiple times with each time counted. Mosquitoes aged 5–10 d were used in bioassays because at this age they show strong attraction toward hosts. During observations, the netting on the top of the container was covered with a plastic wrap sheet to minimize potential interference from exhalation of CO_2_, a known mosquito activator [26]. After the experiment, the glass vials were washed using detergent and then rinsed and dried. Plastic cartons containing the experimental mosquitoes were placed in the freezer until the mosquitoes were dead. The mosquitoes were then removed and the inside of the carton was wiped out using a damp sponge. When experiments (= trials) were repeated, this was always on a separate day with a different batch of mosquitoes. Trials involving more than 1 subject were carried out in the afternoon (between 13:00 and 16:00) and under fluorescent lighting in a classroom setting. Several variations of the basic experiment were carried out over the 5 years of the study and the details are described next.

Experiment 1. In the single 2008 experiment, mosquitoes were placed in cages on the day of the experiment and starved [no sugar or water] for 2–3 h before the trial began. The 26 subjects carried out the pre-ingestion bioassay, and each person then peeled and consumed one standard size (ca. 126 g) banana. The bioassay was repeated at 3 h post-ingestion.

Experiment 2. For the 2009 experiments, mosquitoes were taken off sugar water the night before the experiment, placed in cages on the day of the experiment, and then allowed to acclimate in the testing room for at least 1 h prior to the experiment. Bananas were peeled by two investigators instead of the subjects, and the subjects (N = 26; 11 males, 15 females) were provided with plastic utensils to avoid handling of the fruit. Three trials over three successive weeks, each using an independently reared cohort of mosquitoes, were completed as replicates of the banana experiment. In two additional trials, the subjects did not consume any fruit but maintained the same timing between pre and “post” bioassays (two no-fruit control replicates). This was to determine if mosquito contact rates changed over the course of the afternoon due to extraneous variables (e.g., change in room temperature, humidity, time of day, and change in student skin volatiles). For the final 2009 trial, subjects ingested grapes, again using plastic utensils to avoid contact with the fruit. Each person received 125–130 g of grapes because the average weight of a peeled bananas was 126 g. For all of these experiments, the contact rates were measured at 2 h post-ingestion.

Experiment 3. In 2011, mosquitoes were placed in experimental cages the day before the experiments and the sugar water was replaced with water overnight. Cages were allowed to acclimate to the room for at least 1 h before the trials. We randomized the subjects (N = 20, 9 males, 11 females), so half of the group received grapes and the other half received bananas during the same trial. In a second trial (about one month later), the reciprocal treatment was applied to each individual. The contact assays were performed before and at 1 and 2 h post-ingestion of the fruit.

Experiment 4. In 2011 and 2012, a single student carried out repeated trials to determine the consistency of the banana effect. The student wore disposable gloves to peel the banana and then used a fork for ingestion. Over the course of 15 weeks (15 trials), he measured contact rates at 1 h post-ingestion for *A. stephensi*. On two occasions, he consumed three bananas to determine whether dose mattered. For these experiments, we controlled for variation in mosquito activity patterns by also testing control vials (vials with warm water only, no human substances) at both the pre- and post-ingestion time points.

Experiment 5. In 2012, the same individual carried out five replicates to test the impact of banana ingestion on contact rates at 1 h post-ingestion for *A. gambiae*.

Statistical analysis. We first analyzed results from each year separately. For Experiment 1, the mean number of contacts were compared between treatments (pre-ingestion and post-ingestion bioassays) using a paired Student’s *t*-test. For Experiment 2, data from three banana trials, the single grape trial, and the two trials where no fruit was consumed were combined and analyzed using a repeated measures analysis of variance (ANOVA, SAS Institute, Inc. Cary, NC, USA) and the proc mixed function. For this analysis, the independent variables were treatment, sex, and treatment × sex. Sex was not significant and was removed from the analysis. The dependent variable, change in attractiveness, was expressed as the number of contacts pre-ingestion and the number of contacts at either the 1 or 2 h time points post-ingestion for each individual. Individuals were treated as the repeated factor. No transformations were needed based on the residual plot and the QQ plot showing the normality assumption of the errors was met. Results from Experiment 3 were analyzed in the same way.

Next, a combined dataset was analyzed using a repeated measures ANOVA, constructed using SAS software, Version 9.4, and the proc mixed function. This dataset included the results of mosquito contacts at 1 and 2 h post-ingestion from the multiple experiments in 2009 and 2011. The 2008 data was not included because the only contact measurements occurred at 3 h post-ingestion. No transformations were needed based on the residual plot and the QQ plot showing the normality assumption of the errors was met. Fixed effects were treatment, year, sex, and all first-order interactions. The year in which the experiment took place and the sex of individuals were not significant and were removed from the model along with various non-significant interaction effects. Individuals were treated as the repeated factor.

## 3. Results

From 2008 to 2012, several different groups of subjects carried out pre- and post-ingestion tests and the glass vial bioassay. Because aspects of the procedure were modified and improved over time, we report the results of analysis of each modification separately as well as of a combined dataset.

Experiment 1. There was an average of 1.8 ± 2.5 contacts for the pre-ingestion test compared with 6.8 ± 7.3 contacts 3 h after ingestion of the banana. Of the 26 subjects tested, 16 had an increase in the number of mosquito contacts after eating the banana (mean increase = 8.75), four had a decrease in mosquito contacts after banana consumption (mean decrease = 2.75) and six showed no change. There was a statically significant increase in the mean number of mosquito contacts after banana consumption for this single trial based on a paired Student’s *t*-test (*t* = 3.2937; *df* = 25; *P* = 0.003).

Experiment 2. Six trials over six weeks were completed in 2009. There was little change in contact rates before or after ingestion for the two no-fruit trials, the grape trial, and the first banana trial. However, two of the banana trials showed an effect, with the average number of contacts increasing after banana ingestion by 60 and 40%. We cannot explain the lack of an effect in the first banana trial. However, when the three banana trials were analyzed using a Student’s *t*-test, the overall results were still significant (*t* = 2.6709; *df* = 73; *P* = 0.009). The repeated measures ANOVA for this dataset suggested that treatment affected contact rates (F = 8.81_(3, 71)_; *P* < 0.0001).

Experiment 3. The crossover experiment was completed in two trials. This experimental design provided better control of day-to-day variation in mosquito behavior because the impacts of the ingestion of grapes and of bananas were both tested during the same trial (i.e., the same 2 h time period). The ingestion of bananas was strongly associated with an increase in the number of contacts (F = 4.93_(4, 82)_; *P* = 0.0013) and the effect was significant at both the 1 h (*t* = 3.79; *df* = 86; *P* = 0.0003) and 2 h (*t* = 2.34; *df* = 86; *P* = 0.0214) time points. The estimates for post-ingestion mosquito contacts compared to pre-ingestion showed an increase of ~11 contacts after 1 h and ~7 contacts after 2 h. Ingestion of grapes had no effect (Table 1).

**Figure 1:**
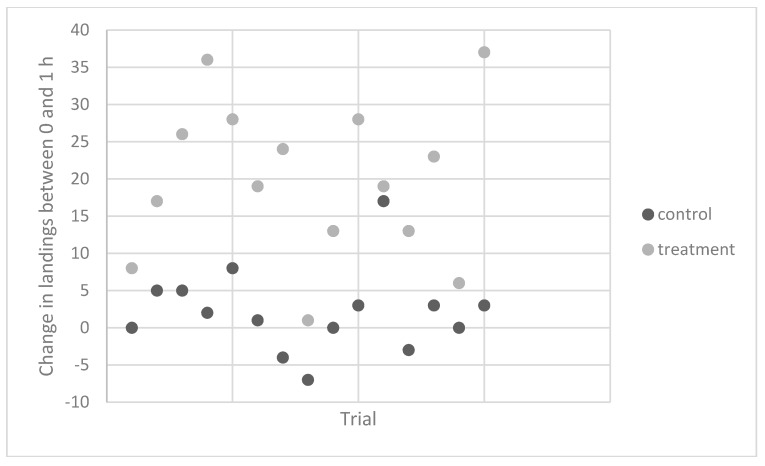
Change in contact counts for *Anopheles stephensi* Liston between 0 and 1 h for control (no rubbing) and treatment (rubbing, banana ingestion) vials. All trials were done using the same individual.

Experiment 4. To determine the consistency of the response, repeated trials with a single individual were performed over 15 weeks in 2011 and 2012. Contact counts were similar for the untreated control vials at the starting time and both the 1 and 2 h times (Table 2, Figure 1). However, the average number of contacts on vials that were rubbed at 1 h after banana ingestion was more than twice as high as those measured for the pre-ingestion vials. For two of these trials, this subject ate three bananas but found no evidence that contact counts were higher than those observed following ingestion of one banana.

**Figure 2:**
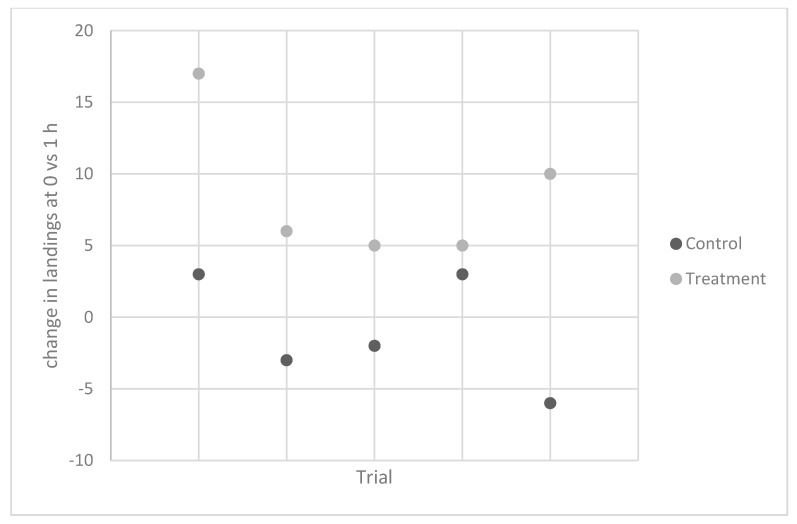
Change in contact counts for *Anopheles gambiae* Giles between 0 and 1 h for control (no rubbing) and treatment (rubbing, banana ingestion) vials. All trials were done using the same individual.

Experiment 5. In 2012, the same individual carried out five trials to test the effects on *A. gambiae*. Again, contact counts were similar for the control vials at the two time points, while there was a 67% increase in contacts measured 1 h after ingestion (Table 3, Figure 2).

Analysis of combined dataset. The combined dataset included 493 observations from 75 individual subjects from experiments concluded from 2009 to 2011. The results reveal that 1 h and 2 h after ingestion of bananas, there was an increase of 12.2 and 6.3 mosquito contacts compared to pre-ingestion contacts, respectively. The average pre-ingestion count was 8.0 mosquito contacts. Thus, the increased contacts after banana ingestion represent an increase of 165% at 1 h and 79% increase at 2 h. Conversely, the ingestion of grapes did not result in a significant difference in number of mosquito contacts compared to pre-ingestion levels. Likewise, there was no significant difference between mosquito contacts at any time for individuals who consumed no fruit.

Despite differences in methodology between years, the analysis of the combined datasets from 2009 and 2011 revealed that the difference between treatment groups was significant. The results of the repeated measures ANOVA showed that the interaction effect between treatment and time was significant, so we considered them simultaneously. At the 2 h time point, the banana treatment group had approximately 11 more mosquito contacts than did the no-banana group (*t* = 7.70; *df* = 47; *P* < 0.0001).

## 4. Discussion

This work provides substantial evidence in support of folk wisdom regarding bananas and mosquito attraction. Over multiple years and with a subject panel of 75 individuals, we demonstrated that ingestion of bananas, but not grapes, resulted in significantly higher attraction of *A. stephensi* at 1, 2, or 3 h after ingestion for some (but not all) of the subjects. This surprising result was confirmed with repeated tests by a single individual, which documented stability and repeatability of the effect over many weeks. Bananas and beer [20,21] are the only dietary components that have been shown to increase mosquito attraction. Digestion of some types of foods appears to result in rapid metabolic changes that alter host odors that are detectable by mosquitoes and may increase the likelihood of contact and biting.

Although the trials were well replicated, the results should be interpreted cautiously. First, the study design relied on contact counts by the subjects themselves before and after food ingestion. It is possible that observer bias affected the counts since the treatment was not blinded. However, the lack of increased contacts following ingestion of grapes and the increased contacts seen in repeated trials by one observer provides significant additional support for the hypothesis. Second, the “banana effect” was observed for two species of anopheline mosquitoes that are native to Africa and Asia. No tests were performed for culicine species, including important vectors such as *Aedes aegypti* (L.), *Ae. albopictus* (Skuse), or *Culex pipiens* (L.), or for other species found in North America. It is likely that host diet affects the attraction of these species as well, but this remains to be tested. Third, the bioassays focused only on substances associated with the human palm. Odors from this part of the body may not be representative of other areas of the body (e.g., legs and arms), which are more likely to be contact sites for mosquitoes. Additional tests that compare biting rates with human subjects and an arm-in-cage design will be needed to validate results. Fourth, the experimental design allowed for measuring attraction only at very close range, and future studies should test whether increases in attraction can be detected over longer distances. Finally, it is important to emphasize that the “banana effect” was not observed for all subjects and there is no way to predict a response for any individual. For these reasons, this work should not be used as the basis for advocating changes in diet.

Bananas are a staple food in many parts of the world, and there are more than 1000 varieties including plantains and other cooking bananas. The glass vial bioassay is a useful screening tool and can be applied to examine responses to these and other foods, including those suggested by the general public as likely to enhance or reduce attractiveness. Future use of the glass vial bioassay could include blinding, perhaps by having a researcher who does not know the treatment rub the vial over the volunteer’s palms and count the number of contacts. In addition to broadening the panel of foods and mosquitoes examined, future studies should also include investigations of the chemical changes resulting in altered attraction. In preliminary studies, we were able to elute the active material from the glass vial onto paper filter discs and to record increased mosquito attraction using the dried discs attached to warm glass vials. Thus, it may be possible to compare chemical profiles for single individuals in pre- and post-ingestion samples using GC/MS [27].

The mechanism underlying the chemical change is also of interest. The data reveal a shift in attractiveness by 1 h post ingestion, which requires a rapidly realized link between digestion and skin secretions. A recent study documented differences in the human plasma metabolome and related these to host attractiveness to *Ae. aegypti* [28]. Other work has shown changes in the plasma metabolome at 1 h following ingestion of bananas [29]. Thus, we speculate that this could be the connection between digestion and skin odors. How the plasma metabolome affects skin odors is not known. Whether these changes enhance mosquito feeding success or reproductive outcomes is also not known.

This work was initiated to gather data in support of outreach and educational materials on ineffective methods for mosquito bite reduction. Instead, we found that there may be an increase in attractiveness following banana ingestion for some people. In that regard, the study served as a reminder that expert advice should always be based on controlled trials. Similar studies should be undertaken to test the claim that personal products, including perfumes, lotions, and shampoos, should be avoided to reduce mosquito bites.

## 5. Conclusions

Two species of malaria vector mosquitoes, *Anopheles stephensi* and *A. gambiae,* demonstrated a significant increase in attraction to human hand secretions 1 h after ingestion of a banana. Results support the hypothesis that diet is an important aspect of mosquito–host interactions.

## Figures and Tables

**Table 1:** Results of the crossover trials (experiment 3).

Treatment	Estimate	SE	DF	*T* Value	Pr > *t*
Banana 1 h	10.8611	2.8631	86	3.79	0.0003
Banana 2 h	6.7111	2.8631	86	2.34	0.0214
Grape 1 h	0.6585	2.5850	86	0.25	0.7995
Grape 2 h	−0.1934	2.5850	86	−0.07	0.9405
Pre-ingestion	0	.	.	.	.

Model. Treatment had a significant effect on contacts. Effect: Num DF (4); Den DF (86); F Value (4.93); Pr > F (0.0013).

**Table 2:** Contact counts for *Anopheles stephensi* Liston before and after banana ingestion using a glass vial bioassay. Results are from repeated trials by a single subject.

Year	Number of Bananas Ingested	Trial	Control^a^ 0 h	Control^a^ 1 h	Rubbed Vial 0 h (Pre-Ingestion)	Rubbed Vial 1 h
2012	1	1	6	6	8	16
2012	1	2	18	23	25	42
2012	1	3	11	16	23	49
2012	1	4	18	20	28	64
2012	1	5	26	34	34	62
2012	3	6	14	15	20	39
2012	3	7	20	16	30	54
2011	1	8	16	9	12	13
2011	1	9	15	15	13	26
2011	1	10	30	33	22	50
2011	1	11	6	23	15	34
2011	1	12	8	5	9	22
2011	1	13	7	10	16	39
2011	1	14	2	2	6	12
2011	1	15	3	6	5	42
		AVE	13.3	15.5	17.7	37.6
		STDEV	8.3	9.7	9.1	16.9

^a^ Control vials were not rubbed but contained body temperature water.

**Table 3:** Number of contacts by female *Anopheles gambiae* Giles before and after ingestion of a single banana. Results are from repeated trials by a single subject.

Trial	Control^a^ 0 h	Control^a^ 1 h	Rubbed Vial 0 h (Pre-Ingestion)	Rubbed Vial 1 h
1	10	13	13	30
2	10	7	13	19
3	3	1	6	11
4	15	18	24	29
5	21	15	33	43
Ave	11.8	10.8	17.8	26.4
STDEV	6.7	6.8	10.7	12.1

^a^ Control vials were not rubbed but contained body temperature water.
